# Absence of Uncoupling Protein-3 at Thermoneutrality Impacts Lipid Handling and Energy Homeostasis in Mice

**DOI:** 10.3390/cells8080916

**Published:** 2019-08-17

**Authors:** Assunta Lombardi, Rosa Anna Busiello, Rita De Matteis, Lillà Lionetti, Sabrina Savarese, Maria Moreno, Alessandra Gentile, Elena Silvestri, Rosalba Senese, Pieter de Lange, Federica Cioffi, Antonia Lanni, Fernando Goglia

**Affiliations:** 1 Department of Biology, University of Naples “Federico II”, 80138 Naples, Italy; 2 Department of Biomolecular Sciences, University of Urbino “Carlo Bo”, 61029 Urbino, Italy; 3 Department of Chemistry and Biology, “Adolfo Zambelli” University of Salerno, 84084 Salerno, Italy; 4 Department of Science and Technology, University of Sannio, 82100 Benevento, Italy; 5 Department of Environmental, Biological and Pharmaceutical Sciences and Technologies, University of Campania “Luigi Vanvitelli”, 81100 Caserta, Italy

**Keywords:** uncoupling protein, mitochondria: energy metabolism, lipid handling, fatty acid oxidation

## Abstract

The role of uncoupling protein-3 (UCP3) in energy and lipid metabolism was investigated. Male wild-type (WT) and UCP3-null (KO) mice that were housed at thermoneutrality (30 °C) were used as the animal model. In KO mice, the ability of skeletal muscle mitochondria to oxidize fatty acids (but not pyruvate or succinate) was reduced. At whole animal level, adult KO mice presented blunted resting metabolic rates, energy expenditure, food intake, and the use of lipids as metabolic substrates. When WT and KO mice were fed with a standard/low-fat diet for 80 days, since weaning, they showed similar weight gain and body composition. Interestingly, KO mice showed lower fat accumulation in visceral adipose tissue and higher ectopic fat accumulation in liver and skeletal muscle. When fed with a high-fat diet for 80 days, since weaning, KO mice showed enhanced energy efficiency and an increased lipid gain (thus leading to a change in body composition between the two genotypes). We conclude that UCP3 plays a role in energy and lipid homeostasis and in preserving lean tissues by lipotoxicity, in mice that were housed at thermoneutrality.

## 1. Introduction

Uncoupling protein-3 (UCP3) is a mitochondrial protein, first discovered in 1997 [1], and prevalently expressed in skeletal muscle (SkM), the heart and brown and white adipose tissues [2]. The extent of homology between the UCP1 and UCP3 genes led to the proposal that UCP3 might be involved in thermogenic mechanisms, and although it does not appear to contribute to cold-induced thermogenesis [3], it has recently proved to be essential in thermogenic responses that are induced by the endotoxin lipopolysaccharide and by the sympathomimetic methamphetamine [4]. Indeed, the evidence that up-regulation of UCP3 is not always associated with mitochondrial uncoupling/thermogenesis [5], which suggested that uncoupling oxidative phosphorylation is not the primary role of UCP3, but rather a consequence of its true function. Other than thermogenesis, other roles that have been attributed to UCP3 include prevention of damage induced by reactive oxygen species (ROS) and lipid hydroperoxides (LOOH), as well as modulation of lipid handling [6,7,8,9]. Indeed, high amounts of UCP3 are present in tissues that are known to metabolize fatty acids (FA) to a high extent, and enhanced levels of UCP3 expression are observed under physiological and pathological conditions, in which the fatty acid oxidation rate is elevated [8,9,10]. In addition, the expression of UCP3 during heart development is correlated to that of mitochondrial fatty acid oxidation rate markers [11]. Furthermore, the absence of UCP3 negatively influences the ability of SkM mitochondria to oxidize FA [12,13]. In this context, Bouillaud et al. [14] suggested that UCP3 could switch cells from carbohydrate to fatty acid metabolic pathways by promoting mitochondrial pyruvate extrusion, which prevents the use of pyruvate as a substrate. However, mechanistic information on this possible activity is currently lacking.

Although the roles that were proposed for UCP3 suggest that it could play a role in energy homeostasis (EH), the obtained contrasting results have not produced a common and unambiguous conclusion so far. Several lines of experimental evidence supporting the role of UCP3 in EH came from human studies [see 6 and references within] and from mice over-expressing UCP3, being: (i) obesity-resistant mice present higher UCP3 levels than obesity-prone mice [15]; (ii) transgenic mice that over-express UCP3 are metabolically less efficient than their wild-type litter mates, and are protected against high fat diet (HFD)-induced obesity [16,17]; and, (iii) modest UCP3 over-expression in SkM increased mice energy expenditure [18]. Conversely, some studies that were performed on mice lacking UCP3 (KO mice) are discordant, since they did not show alterations in several metabolic parameters, such as the resting metabolic rate, the regulation of body temperature during cold exposure, food intake, body weight regulation, and the total body triglyceride content [3,19]. Nevertheless, KO mice have been shown not to be obese [3] (only showing higher lipid accumulation relative to WT litter mates when fed a HFD for a prolonged period (eight months)). Furthermore, the KO mice showed no alteration in metabolic efficiency [20]. The absence of metabolic alterations in KO mice could be due to the constant thermal stress that is caused by the animal housing conditions. In fact, in most studies, mice (which have a thermoneutrality temperature of 30 °C) are housed at the standard temperature (20–24 °C), which represents cold stress. Thus, mice lacking UCP3 that are exposed to thermal stress may implement compensatory mechanisms to maintain their body temperature, and these mechanisms are likely to affect the overall metabolic rate and other investigated parameters. Such a possibility was previously tested for UCP1 [21]. UCP1 ablation only induced obesity when the mice were housed under thermoneutral conditions. This outcome highlights the importance of avoiding thermal stress in metabolic studies on mice, since housing temperatures significantly influence the outcome of experiments, as well as their translatability to humans that, indeed, create a thermoneutral environment without cold stress for themselves [22]. Here, we investigated the role of UCP3 in metabolic control in situations in which thermal stress was eliminated. We report that, in adult mice acclimated at thermoneutrality for two weeks, UCP3 ablation altered energy and lipid metabolism. Moreover, in standard diet fed mice, which were kept at thermoneutrality since weaning, UCP3 ablation enhanced ectopic fat accumulation in liver and skeletal muscle, and vastly augmented HFD-induced fat accumulation. We conclude that the exposure temperature is determinative for the outcome of metabolic effects elicited by UCP3 and that the protein can be involved in the metabolic control of lipid metabolism in mice and possibly in humans.

## 2. Materials and Methods

### 2.1. Materials

All of the chemicals were purchased from Sigma-Aldrich (St. Louis, MO, USA), unless otherwise specified.

### 2.2. Animals

UCP3-ablated mice were derived from those that were described by Gong et al. [3] and they were backcrossed to the C57BL/6 strain for ten generations.

Male wild type (WT) and UCP3 knockout mice (KO) were used in the present study. Mice were housed in thermoneutrality (30 ± 1 °C) with a 12/12 h light-dark cycle and free access to food and water. This study was carried out in accordance with recommendations in the EU Directive 2010/63/ for the Care and Use of Laboratory Animals. The Committee on the Ethics of Animal Experiments of the University of Napoli Federico II (Italy) and the Italian Minister of Health approved all of the animal protocols. Every effort was made to minimize animal pain and suffering. At the end of the treatments described below, the mice were anesthetized with Zoletil (40 mg/100 g bw) and sacrificed by decapitation.

To detect metabolic parameters and mitochondrial functionality, four–five month-old mice of each genotype acclimated to 30 °C ± 1 for 2–3 weeks and fed a standard diet were used to detect resting metabolic rate (RMR), respiratory quotient (RQ), energy expenditure (EE), and mitochondrial functional parameters. Other groups of WT and KO mice were kept one per cage and were housed at 30 °C ± 1 for 80 days since weaning and fed with a standard diet or a high fat diet (HFD) for 80 days in order to detect body composition and energy gains as well as to perform histological analysis of mice under different “lipid loads‘’. Standard/low fat diet (STD) consisted of 10% lipids, 20% proteins, 70% carbohydrates with a gross energy density of 15.5 kJ/g wet food. HFD consisted of 45% lipid, 20% proteins, and 35% carbohydrates with a gross energy density of 19.2 kJ/g wet food. Both of the diets were from Mucedola (Milano Italy).

### 2.3. Metabolic Parameters

Oxygen consumption (VO_2_) and carbon dioxide production (CO_2_) measurements were made using a four-chamber, indirect open-circuit calorimeter (Columbus Instrument), with one mouse per chamber at a room temperature of 30 °C to evaluate basal metabolic parameters. Measurements were performed between 1100 and 1600 h. After a 1-h period of adaptation to the metabolic chamber, VO_2_ and VCO_2_ were measured when the mice were not moving for at least 10 min. The system settings included a flow rate of 0.5 L/min., a sample line-purge time of 2 min., and a measurement period of 30 s every 12 min. Mice were placed in separate 2.5-L calorimetry chamber with ad libitum access to water. Values of VO_2_ and VCO_2_ were obtained by means of three different consecutive measurements during which the mice were not moving. These data were used to calculate the respiratory quotient (RQ; VCO_2_/VO_2_) and the resting energy expenditure (REE) ([3.815 + 1.232 RQ] VO_2_). The contribution of fatty acid oxidation to REE was calculated, as described using the following equation: percentage of fat contribution = [468.6 (1 − RQ)]/[5.047 (RQ − 0.707) + 4.686 (1 − RQ)] [23].

### 2.4. SkM Mitochondrial Respiration

As soon as euthanasia was performed, SkM were excised and all visible contaminating tissues were removed. The tissues were either immediately processed for mitochondria isolation or frozen in liquid nitrogen and then stored at −80 °C for later analysis. Mitochondria from SkM were isolated by differential centrifugation, as reported by Lombardi et al. [24]. The mitochondrial respiration and fatty acid oxidation rate were assessed by the polarographic method while using a Clark-type electrode at 37 °C by using different respiratory substrates. SkM mitochondrial respiration was detected in a final volume of a 0.5 mL respiration medium consisting of 80 mM KCl, 50 mM HEPES (pH 7.0), 1 mM EGTA, 5 mM K2HPO4, and 0.5% fatty acid-free BSA (w/v). The mitochondria were incubated for three min in the respiratory medium, and the respiration was initiated by the addition of succinate (5 mM) in the presence of rotenone (4 µM) or pyruvate (10 mM) in the presence of malate (2 mM), or palmitoyl-carnitine (40 µM) in the presence of malate (2 mM). Once State 2 of respiration was reached, ADP (300 µM) was added to the incubation medium to induce State 3 respiration; when ADP was exhausted, State 4 was reached.

### 2.5. Separation of Respiratory Complexes by Blue-Native Page (BN-PAGE) and Histochemical Staining for in-Gel Activity

Solubilisation of mitochondrial membranes by detergents, BN-PAGE, staining, and densitometric quantification of oxidative phosphorylation complexes were performed, as described in Scagger et al. [25] and Lombardi et al. [26], with some minor variations. Mitochondria enriched fraction was suspended in a low-salt buffer (50 mM NaCl, 50 mM imidazole, pH 7.0) and solubilised with 10% (w/v) dodecyl-maltoside to solubilise the individual respiratory chain complexes. The electrophoretic run was carried out on 4–13% gradient polyacrylamide gels and enzymatic colorimetric reactions were performed essentially as reported by Zerbetto et al. [27]. The activity of complex I activity was evaluated by incubating the gel slices with 2 mM Tris–HCl, pH 7.4, 0.1 mg/mL NADH, and 2.5 mg/mL nitro blue tetrazolium (NTB) at room temperature. To detect complex II activity, gel slices were incubated at room temperature in a 100 mM Tris/glycine buffer at pH 7.4 containing 1 mg/mL NTB and 1 mM sodium succinate. Complex IV activity was assessed by incubating BN-PAGE gels with 5 mg 3,3′-diaminobenzidine tetrahydrochloride (DAB) that was dissolved in a 9 mL phosphate buffer (0.05 M, pH 7.4), 1 mL catalase (20 μg/mL), 10 mg cytochrome c, and 750 mg sucrose. The original colour of the complex I, II, or IV-reacting bands was preserved by fixing the gels in 50% methanol and 10% acetic acid. In parallel, another electrophoretic run was performed to stain the gels with Coomassie Blue G to obtain the total band pattern of the respiratory complexes. After gel scanning, the areas of the bands were expressed as absolute values (arbitrary units).

### 2.6. Determination of Glycerol Release from White Adipose Tissue

100 mg of epididymal white adipose tissue samples were removed. Samples were cut into 20 mg sections to evaluate the glycerol diffusion from tissue to the medium better; 100 mg of tissue were incubated at 37 °C in 500 μL of Krebs Ringer buffer (KRB; 12 mM HEPES, 121 mM NaCl, 4.9 mM KCl, 1.2 mM MgSO_4_, 0.33 mM CaCl_2_) containing 2% FA-free bovine serum albumin (BSA) and 0.1% glucose in the presence or absence of 10 μM isoproterenol (Sigma). Tissue was incubated for 1 h at 37 °C in a shaking bath and then gassed with 95% O_2_-5% CO_2_. At the end of the incubation period, an aliquot of the medium was used for the analysis of glycerol. A commercially available absorbance-based enzyme assay for glycerol (Free Glycerol Reagent; Sigma) was converted to fluorescence-based detection by the inclusion of the hydrogen peroxide-sensitive dye Amplex UltraRed, as reported by Clark et al. [28].

### 2.7. Hystological Analysis

Samples of visceral WAT (mesenteral), liver, and gastrocnemius skeletal muscle were fixed by immersion in 4% formaldehyde in 0.1 M phosphate buffer (overnight at 4 °C). The samples were dehydrated in ethanol, cleared, and then embedded in paraffin blocks. The tissues were cut into serial 6-πm-thick sections and then stained with hematoxylin-eosin for morphological examination. For adipocyte size quantification, evaluations were performed on three different hematoxylin-eosin slides (sections every 400 µm) for each animal and at least 400 adipocytes per animal were analyzed. The sections were viewed with a Nikon Eclipse 80i light microscope (Nikon Instruments, Milan, Italy) at 20× magnification. Images were obtained with a Sony DS-5M camera connected to an ACT-2U image analyzer. The mean surface area and the frequency distribution were calculated from at least four mice for each group, adipocyte size distribution is presented as the percentage of the total amount of cells.

### 2.8. Body Composition and Energy Gains

Body composition and carcass energy content were evaluated, as reported by Iossa et al. [29]. In brief, after removing the gut contents, the carcasses were autoclaved and homogenized in water. The water content of the carcass was detected by drying the homogenate at 70 °C in a vacuum oven. Small pellets of dried homogenate were then used to evaluate the carcass energy content by bomb calorimetry. Other homogenate aliquots were used to detect the lipid content by the Folch et al. method [30]. Body protein content was determined from a general formula relating to the total energy value of the carcass, the energy derived from fat, and the energy derived from protein [31,32]. The caloric values for body fat and protein were taken as 38.6 and 22.7 kJ/g, respectively. To detect lipid and protein gains, as well as energy efficiency in WT and UCP3 KO mice, six mice from each genotype were euthanatized at weaning (i.e., when they were 24 days old) that corresponded to the beginning of dietary treatments (groups were named WT-time 0 and KO-time 0). Two additional groups of mice for each genotype were individually caged for 80 days since weaning, and feed ad libitum with either a STD diet or a HFD, as described above (groups were named WT-STD, KO-STD, WT-HFD, KO-HFD). The duration of the treatment with the high fat diet was chosen, since it was long enough to induce a HFD induced obesity in mice that were acclimated at thermoneutrality [33]. During the treatment, the body weight and food intake of the mice were monitored twice weekly. Feed spillage was taken into account when calculating the energy intake during the treatment. Body composition and energy content were evaluated, as described above. Carcass total energy gain, as well as the amount of energy that is gained and stored as lipids or as proteins after 80 days of either STD or HFD were determined by subtracting the total carcass energy, the carcass lipid-derived energy, and the carcass protein-derived energy detected in the WT-time 0 and KO-time 0 groups from the respective values detected in the WT-STD, WT-STD, KO-STD, and KO-HFD groups. The efficiencies of energy, lipid, and protein deposition were calculated as: (energy gain/energy consumed by diet) × 100, (lipid gain/lipid-derived energy consumed by diet) × 100, (protein gain/protein-derived energy consumed by diet) × 100, respectively.

### 2.9. Statistical Analysis

Data are reported as mean ± SEM and have been analyzed by Student’s *t*-test or by two-way ANOVA, followed by Tukey’s post-hoc test. Analyses have been performed with Graphpad Prism 5 software. Differences have been considered to be statistically significant when *p* < 0.05.

## 3. Results

### 3.1. UCP3 Ablation Affects Resting Metabolic Rate, Energy Expenditure, and Fatty Acid Utilization in Adult Mice Acclimated at Thermoneutrality

**Figure 1:**
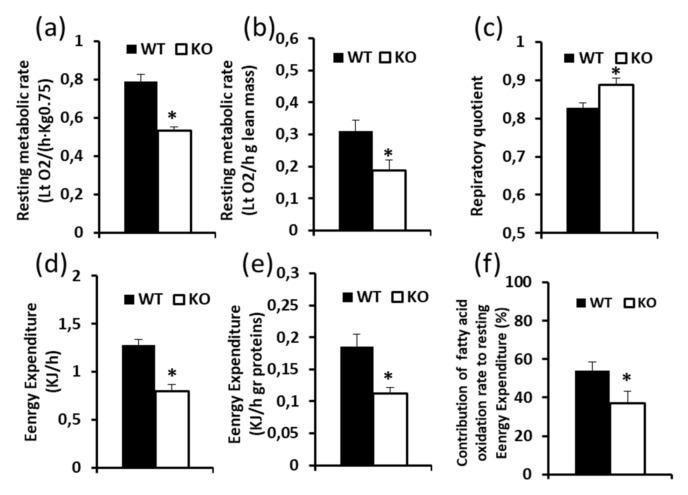
Effect of UCP3 ablation on metabolic parameters detected in WT and KO mice housed at thermoneutrality for 2–3 weeks and fed a standard diet (**a**), (**b**) Resting Metabolic Rate, (**c**) Respiratory Quotient, (**d**), (**e**) Energy Expenditure, (**f**) contribution of fatty acid oxidation to energy expenditure (EE). Values represent mean ± SE of 6–7 animals for WT and KO mice, respectively. Statistical analyses were performed by two-tailed Student’s *T*-test, * *p* < 0.05 vs. WT.

The resting metabolic rate (RMR), the resting energy expenditure (REE), and the respiratory quotient (RQ) were detected in 4–5 month old animals, which were acclimated at thermoneutrality for at least two weeks. RMR was significantly reduced in KO mice as compared to WT mice (−30%) (Figure 1), both when it was expressed in Litres oxygen/(hour Kg^0.75^) and when expressed in Litres oxygen/(hour g of lean mass). The RQ was increased in KO mice, which indicates a lower use of lipids as metabolic substrates in these animals. Indeed, in KO mice, the contribution of fatty acid oxidation to REE was reduced by about 27% relative to that in WT mice (Figure 1). In addition, in adult KO animals, food intake resulted in a reduction of about 15%, being the values 3.1 ± 0.11 and 2.63 ± 0.014 g food/day, for WT and KO mice, respectively (*n* = 6, *p* < 0.05 by Student’s *t*-test).

**Figure 2:**
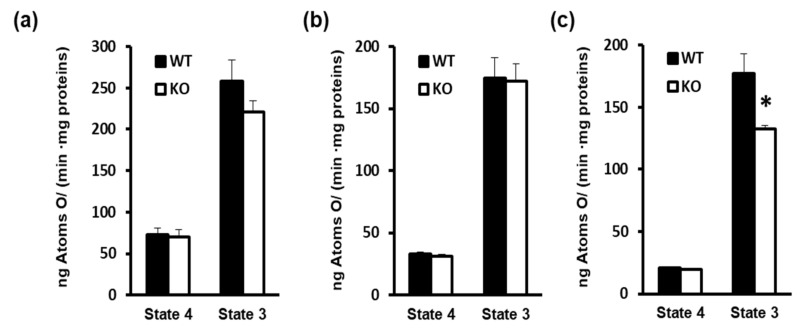
Impact of UCP3 ablation on mitochondrial respiration rate, detected in the presence of different substrates: (**a**) Succinate (+rotenone), (**b**) Pyruvate (+malate), and (**c**) Palmitoyl-carnitine (+malate). Skeletal muscle mitochondria were isolated from WT and KO mice housed at thermoneutrality for 2–3 weeks and fed a standard diet. Values represent mean ± SE of six different animals. Statistical analyses were performed by two-tailed Student’s *t*-test, * *p* < 0.05 vs. WT.

SkM mitochondria that were isolated from WT and KO mice did not show significant differences in respiratory parameters (State 4 and State 3) when using pyruvate + malate or succinate + rotenone as substrate (Figure 2a,b). On the other hand, a significant State 3 inhibition was observed in mitochondria from KO mice when palmitoyl carnitine + malate was used as the substrate, which thus indicated the lower ability of mitochondria to oxidize fatty acids (Figure 2c).

**Figure 3:**
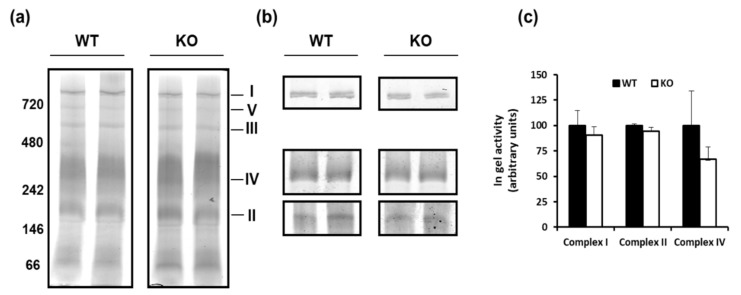
BN-PAGE-based analysis of individual respiratory complexes from dodecylmaltoside-solubilized crude mitochondria from SkM of WT and KO mice housed at thermoneutrality for 2–3 week and fed a standard diet. (**a**) Panels show representative images of Coomassie blue stained BN-PAGE gels. (**b**) Panels show representative images of histochemical staining of complex I (**I**), complex IV (**IV**), and complex II (**II**) in-gel activity. (**c**) Densitomentric quantification of band corresponding to in gel-activity of complex I, complex IV, and complex II. Protein extracts were prepared for each animal, and each individual was separately assessed. Data were normalized to the value obtained for WT animals, set as 100, and separately presented for each genotype (means ± SE; *n* = 3).

Subsequently, we evaluated whether UCP3 ablation could influence respiratory chain complexes activity. In-gel activity of each individual respiratory complex (I, II, and IV) did not differ between WT and KO mitochondria (Figure 3). In view of the above results, we wondered whether UCP3 ablation would affect body fat accumulation in mice that were housed at thermoneutrality since weaning by determining their metabolic phenotype after feeding for 80 days, either with a standard/low fat diet (STD) or a high fat diet (HFD).

### 3.2. Effect of UCP3 Ablation on Body Weight, Energy Efficiency, and Body Composition in Mice Fed Either with a Standard Low Fat Diet or a High Fat Diet

At weaning, representing the experimental starting condition, KO-time 0 mice tended to have a lower body weight when compared to WT-time 0. However, the two groups showed similar body composition in terms of water, lipid, and protein percentage, as well as in energy content, referring to each gram of animal (Table 1).

WT and KO mice housed at thermoneutrality and fed a standard/low fat diet (STD) for 80 days since weaning (named WT-STD and KO-STD, respectively) gained the same body weight, while consuming the same amount of energy by food (Table 2), thus no differences were observed in the rough energy efficiency between WT-STD and KO-STD (Table 2).

**Figure 4:**
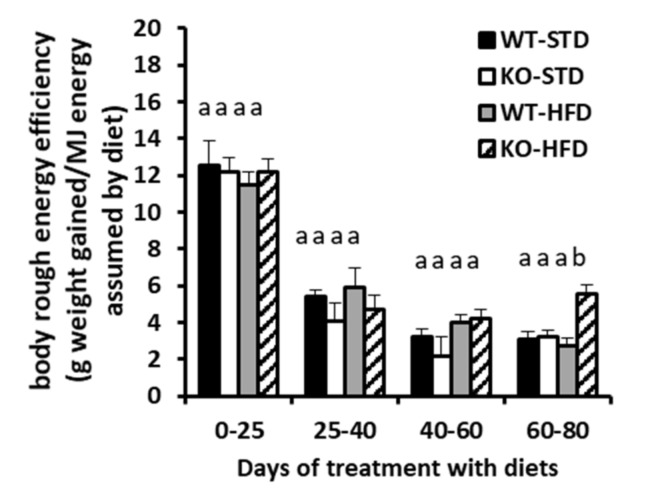
Variation in body rough metabolic energy efficiency during the 80 days of treatment of WT and KO mice with either STD or HFD. Values represent the mean ± SE of 7–8 different animals. The differences were evaluated for statistical significance by two-way ANOVA test, followed by a Tukey’s post hoc multiple comparison test. Different letters indicate that differences between mean values are statistically significant, with *p* < 0.05. Two way ANOVA test results: diet effect *p* = 0.0162, genotype effect *p* = 0.0037, interaction *p* = 0.0081.

As expected, mice that were fed a HFD for 80 days since weaning (named WT-HFD and KO-HFD) gained more body weight when compared to mice under STD feeding, independent of the genotype. Body weight gain tended to be higher in KO-HFD mice than WT-HFD mice, although the food that was consumed was similar between the two groups, thus indicating that WT-HFD-mice have lower body rough energy efficiency than KO-HFD ones (Table 2). Indeed, concerning the last parameter, a two-way ANOVA test indicated the effect of the genotype and that of the diet was not statistically significant, while a significant interaction between genotype and diet was observed (see Table 2 legend). Remarkably, in both groups of mice, body rough energy efficiency changed during the treatment with either STD or HFD. Indeed, it was maximal during the first 25 days and then progressively declined (Figure 4). A difference in rough body energy efficiency between WT-HFD and KO-HFD mice was principally observed at the end of treatment, and, also in this case, a two-way ANOVA test indicated a significant interaction effect between genotype and diet (Figure 4 legend).

**Figure 5:**
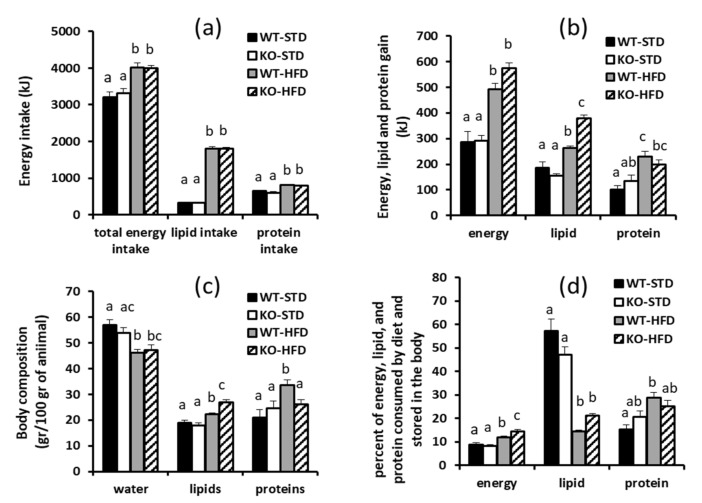
(**a**) Total energy, lipid and protein consumed by diet (**b**) Body energy, lipid and protein gains (**c**) Body composition (**d**) Efficiency of energy, lipid, and protein deposition of WT and KO mice, housed at thermoneutrality, and fed with either a standard/low fat diet (STD) or a high fat diet (HFD) for 80 days since weaning. Body composition was detected in terms of water, lipids, and proteins percentage. Values represent mean ± SE of 5–6 different animals. Differences were evaluated for statistical significance by two-way ANOVA test followed by a Tukey’s post hoc multiple comparison test. Different letters indicate that differences between mean values are statistically significant, with *p* < 0.05. Two way ANOVA test results: - Panel a, for each parameter considered: diet effect *p* < 0.0001, genotype effect ns, interaction ns. - Panel b, energy gain: diet effect *p* < 0.0001, genotype effect ns interaction ns -lipid gain: diet effect *p* < 0.0001, genotype effect *p* = 0.0074, interaction *p* < 0.0001 -protein gain: diet effect *p* < 0.0001, genotype effect ns, interaction ns, Panel c—water: diet effect *p* < 0.0001, genotype effect ns interaction ns -lipids: diet effect *p* < 0.0001, genotype effect *p* = 0.0040, interaction *p* < 0.0032 - protein gain: diet effect *p* = 0.0154, genotype effect ns, interaction ns (*p* = 0.0516), Panel d – Efficiency of energy deposition: diet effect *p* < 0.0001, genotype effect ns (*p* = 0.0913), interaction *p* = 0.013 - Efficiency of lipid deposition: diet effect *p* < 0.0001, genotype effect ns, interaction *p* = 0.0118 - Efficiency of protein deposition: diet effect *p* = 0.0016 genotype effect ns, interaction ns.

We then detected body energy, lipid, and protein gains, as well as the efficiency of energy, lipid, and protein deposition. Concerning energy intake (Figure 5a) and whole body energy gain (Figure 5b), no significant differences were observed between WT-STD and KO-STD. HFD feeding induced a significant increase in mouse energy gain, and, despite KO-HFD tending to have higher body energy gains when compared to WT-HFD, statistical analysis revealed a significant effect of the diet, while the genotype effect and genotype/diet interaction were not significant. When looking at the lipid gain (Figure 5b), similar values were detected in WT-STD and KO-STD. As expected, the HFD regimen induced a significant increase in mice lipid gain, which was significantly higher in KO-HFD when compared to WT-HFD. Indeed, the two-way ANOVA test revealed the significant effects of diet, genotype, as well as the interaction between genotype and diet (Figure 5 legend). No differences between WT-STD and KO-STD were observed regarding protein gain (Figure 5b). The HFD regimen significantly enhanced protein gain, and similar values were detected in WT-HFD and KO-HFD mice (Figure 5b). In fact, the two-way ANOVA test revealed the significant effect of the diet but not of genotype or genotype/diet interactions (Figure 5 legend).

In mice that were fed a STD, the efficiency of the energy deposition (evaluated as percent of energy consumed by diet and stored in the body) was similar in WT and KO mice (Figure 5d). The HFD regimen induced a significant increase in the efficiency of the energy deposition, which was significantly higher in KO-HFD as compared to WT-HFD. Indeed, the two-way ANOVA test revealed the significant effects of diet and the interaction between genotype and diet (Figure 5 legend).

The percentage of energy that was consumed by diet as lipids that was stored in the body did not differ between WT-STD and KO-STD (Figure 5d). HFD feeding significantly reduced such a parameter and, despite KO-HFD mice tending to have higher lipid deposition efficiency than WT-HFD (+40%), differences between these two groups did not reach statistical significance. Concerning the efficiency of protein deposition, no difference was observed between WT-STD and KO-STD. The HFD regimen enhances the efficiency of protein deposition and similar values were detected in WT-HFD and KO-HFD (Figure 5d). When considering either lipids or protein deposition (Figure 5d), two-way ANOVA tests revealed a significant effect of the diet, while neither the effect of genotype or the interaction between genotype and diet were significant (Figure 5 legend).

Consistent with what is described above, after 80 days of STD no differences in mouse body composition (evaluated in terms of content of water, lipids and protein in the carcass and expressed in percentage terms) were detected between WT-STD and KO-STD (Figure 5c). As expected, HFD treatment significantly affected mouse body composition, and a reduction in water content was observed independent of the genotype. Regarding the body lipid percentage, HFD treatment significantly enhanced it, with KO-HFD showing values that were higher than WT-HFD. In this case, the two-way ANOVA test reported the significant effects of diet and genotype, as well as a significant interaction effect between diet and genotype (Figure 5 legend). Concerning the body protein percentage (Figure 5c), HFD treatment enhanced it in WT mice, but failed to affect it in KO mice. Indeed, in the WT-HFD mice the protein percentage was higher than in the WT-STD ones, while no differences between KO-HFD and KO-STD were detected. Two-way ANOVA tests revealed a significant diet effect, being that the genotype effect was not significant and the interaction between the diet and genotype effects were at the limit of significance (*p* = 0.0516).

### 3.3. Effect of UCP3 Ablation on Visceral Adipose Tissue and Lipid Accumulation in Lean Tissue in Mice Fed Either with a Standard/Low Fat Diet or a High Fat Diet

The contribution of metabolically active tissues (liver, heart, gastrocnemius skeletal muscle, brown adipose tissue) to the weight of mice was similar when comparing the WT-STD, KO-STD, WT-HFD, and KO-HFD mice (data not shown). Interestingly, the contribution of total visceral WAT (vWAT) to body weight was lower in KO-STD than in WT-STD mice (about −25%) (Table 2). As expected, in mice under HFD, the contribution of vWAT weight to whole body animal was significantly higher than in mice under STD. Indeed, in WT-HFD the contribution was 56% higher than that of WT-STD, while in KO-HFD it was 97% higher than that observed in KO-STD (Table 2). In this case, a two-way ANOVA test indicated the genotype effect and the diet one as significant, but the interaction effect between genotype and diet as not significant (Table 2 legend).

**Figure 6:**
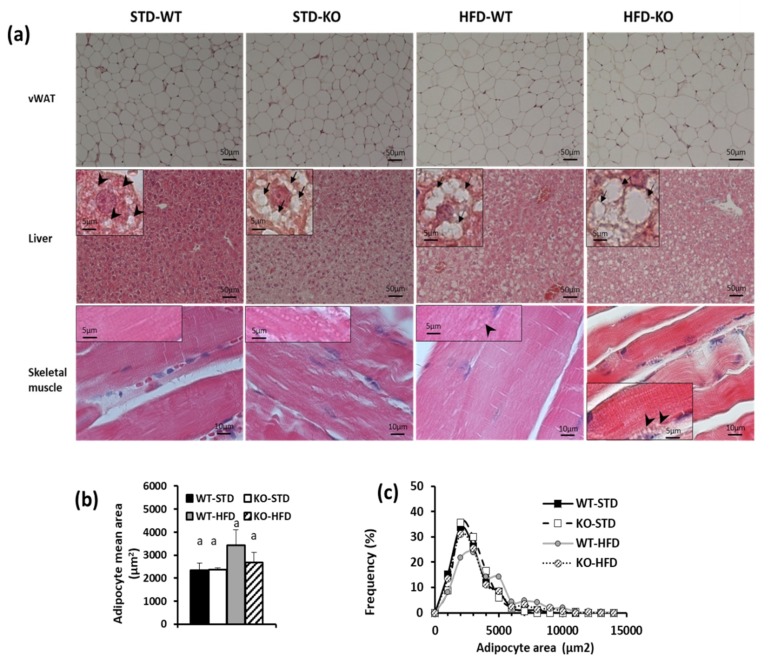
Effect of UCP3 ablation on lipid accumulation in WT and KO mice housed at thermoneutrality and fed with either a standard/low fat diet (STD) or high fat diet (HFD) for 80 days, since weaning. (**a**) Representative histological analysis of visceral WAT, liver and gastrocnemius skeletal muscle isolated from WT and KO mice, housed at thermoneutrality since weaning and fed with a standard/low fat diet for 80 days. Insets: high magnification images (100x) to visualize intracellular LD as uncoloured circles. In liver, arrowheads indicated small LD whereas arrows showed very large LD. (**b**) Mean Surface Area of adipocytes (µm^2^), (**c**) frequency distribution for surface area of adipocyte Values represent mean ± SE of 3 different animals. Differences were evaluated for statistical significance by two-way ANOVA test followed by a Tukey’s post hoc multiple comparison test. Different letters indicate that differences between mean values are statistically significant, with *p* < 0.05. Two-way ANOVA test results for adipocyte mean area: diet effect, genotype effect and interaction ns.

**Figure 7:**
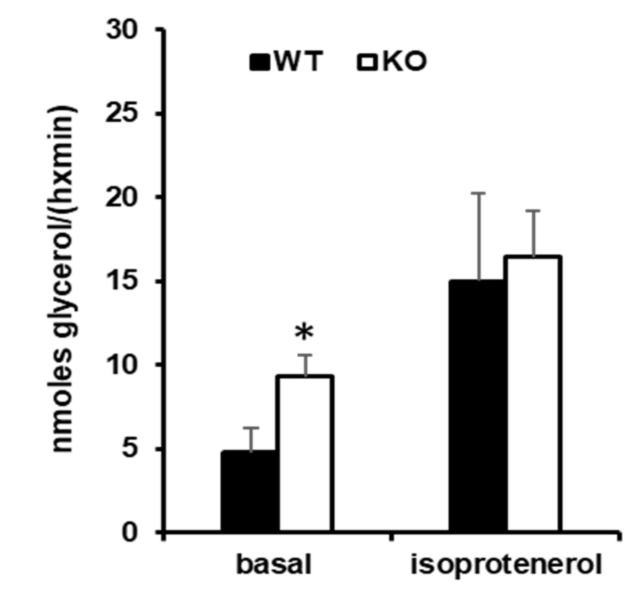
Effect of UCP3 ablation on visceral WAT lipolysis detected in WT and KO mice housed at thermoneutrality and fed with a standard/low fat diet (STD) for 80 days, since weaning. A glycerol release was detected on small pieces of epididymal WAT from WT-STD and KO-STD mice in the absence and in the presence of isoprotenerol. Values represent mean ± SE of 6 different animals. Statistical analyses were performed by two-tailed Student’s *t*-test, * *p* < 0.05 vs. WT.

Variations in vWAT mass, which were detected between WT-STD and KO-STD, were not associated with change in adipocytes size (Figure 6a–c), while WAT basal lipolysis was almost doubled in KO mice as compared to WT (Figure 7). Histological analysis of lean tissues (liver and skeletal muscle) revealed the presence of some lipid droplets in liver from WT-STD exposed at thermoneutrality, in accordance with data present in literature. Interestingly, the same analysis indicated an ectopic accumulation of fat that is associated with the absence of UCP3 in liver and skeletal muscle (Figure 6a). Indeed, H&E staining of skeletal muscle and liver sections from KO-STD at 100× magnification showed many large intracellular lipid droplets (LD) as uncoloured circles. Numerous large (~2 µm) intramyocellular lipid droplets (IMLDs) were only present in the skeletal muscle of KO-STD mice as well as a massive lipid accumulation being shown in the cytoplasm of all the hepatocytes. Mice under HFD presented an accumulation of lipids in liver whatever the genotype, and lipid accumulation in skeletal muscle was more evident in the KO-HFD mice then in the WT-HFD ones.

## 4. Discussion

In this study, we minimized thermal stress to better define the role of UCP3 in whole energy homeostasis and lipid utilization and we showed that in 4–5 month old mice acclimated for 2–3 weeks at thermoneutrality, the absence of UCP3 significantly depresses the whole animal RMR and REE, the energy intake, and the use of lipids as metabolic substrates. The last effect seems to be the result of the impaired ability of SkM mitochondria to oxidize fatty acid, which confirmed the results of previous studies employing mice that were housed at a standard temperature [11,12,34].

Interestingly, the absence of UCP3 selectively reduces the oxidation of lipid derived substrates, such as palmitoyl carnitine (State 3), while not affecting respiratory parameters (both State 4 and State 3) when pyruvate or succinate was used as substrate, i.e., a complex I- linked substrate and complex II-linked substrate, respectively. It should be considered that the control of State 4 respiration is shared between proton leak and reactions that are involved in the oxidation of substrates (among these respiratory chain). The control of State 3 respiration is shared between the reaction involved in the synthesis and export of ATP and the reaction involved in the oxidation of a substrate [35]. Thus, the absence of variations in State 4 respiration suggest that UCP3 does not impact the respiration that is needed to balance the proton leak in basal condition. These data are in line with some studies showing no differences in basal proton-leak kinetics between WT and KO in skeletal muscle mitochondria from mice that were housed at a standard temperature [36,37] and from mice housed at thermoneutrality (our unpublished observation). They also support the concept that UCP3 could only mediate the proton leak in the presence of activators, plausibly ROS and FA [6,37]. At the same time, we did not observe any variations in State 3, in the presence of either pyruvate or succinate, between WT and KO SkM mitochondria. This finding suggests that the absence of UCP3 does not influence the activities of the reactions that are involved in the synthesis and export of ATP, and that are involved in the oxidation of the above substrates, among these the respiratory chain. The functional data, obtained in isolated mitochondria that are oxidizing pyruvate or succinate, agree with the in gel respiratory complexes activity. The reduced state 3, detected in KO mice mitochondria in the presence of palmitoyl-carnitine, suggests that it is plausibly due to the impaired activity of the reactions that are involved in the oxidation of such a substrate.

Data that were obtained in adult animals acclimated to thermoneutrality for two to three weeks suggest that the absence of UCP3 could influence body lipid accumulation. We tested this hypothesis by chronically exposing mice to different lipid loads when housed at thermoneutrality since weaning.

The absence of UCP3 leads mice to be more susceptible to whole animal lipid accumulation only when they are subject to a HFD lipid overload. In fact, HFD-KO mice showed enhanced body energy efficiency and increased body lipid gain, which, by the end of the treatment, produced a different body composition with KO-HFD mice presenting a higher body lipid percentage and lower protein percentage when compared to HDF-WT mice. Interestingly, the evidence that KO-HFD and HFD-WT mice showed the same energy/lipid intake, but at the same time HFD-KO mice accumulated more lipid, further sustains that the absence of UCP3 reduced fat utilization and increased whole body fat storage. As a whole our data also indicates that lipids overload, as obtained by HFD administration, is important to shed light on the involvement of UCP3 in influencing body composition, since, when mice were fed with a standard/low fat diet, the absence of UCP3 does not influence the whole body lipid accumulation as well as body composition.

The ability of KO mice fed a HFD to accumulate more fat than WT mice was previously demonstrated by Costford et al. [15]. However, this accumulation was observed in mice that were housed at 23 °C that had received HFD for eight months, whereas the treatment was ineffective after only four months of HFD [19]. Notably, our data indicates that, when housing mice under thermoneutral conditions, 80 days (~2.5 month) of HFD was enough to produce a higher lipid gain in KO mice when compared to wild type mice. This finding further supports the critical role of housing temperature for metabolic studies. Interestingly, despite the fact that, in young mice fed with standard/low fat diet, the absence of UCP3 does not change whole body lipid accumulation, redistribution of fat in the body does get affected, since it represses lipid storage in visceral WAT, while promoting accumulation in the liver and skeletal muscle.

Previous studies that were performed on KO mice housed at standard temperature and fed with low fat/standard diet failed to show an increase in intramuscular lipid accumulation. This was only observed when KO mice were fed with a HFD for eight months [15], thus further confirming the importance of the housing temperature for the outcome of this phenomenon in the absence of chronic lipids overload.

It should be considered that, in mice, the inability to adequately promote fatty acid utilization is associated with lipid accumulation in peripheral tissues and it contributes to the development of insulin resistance [38], a condition that is more evident when associated with enhanced WAT lipolysis. This “metabolic picture” is observed in KO mice that were acclimated to thermoneutrality, in which fatty acid oxidation is blunted, release of fatty acids by visceral adipose tissue’ depots is enhanced, and the accumulation of lipid in lean tissues (liver and skeletal muscle) takes place. These data suggest that: (i) an alteration in UCP3 activity can also affect the metabolism of tissues that do not express it, such as liver, presumably through changes in systemic metabolite trafficking, and (ii) UCP3 exerts a protective role against lean tissue “lipotoxicity” and insulin resistance, by avoiding ectopic fat accumulation.

The above data are in agreement with our previous studies indicating that a progressive decline in insulin sensitivity in UCP3+/− heterozygous mice and UCP3−/− mice [11] and with clinical observations reporting that (i) a 50% reduction of UCP3 protein in human SkM is correlated with the incidence of T2DM [39], (ii) in humans, the UCP3 protein levels are reduced in the pre-diabetic state of impaired glucose tolerance [40,41], (iii) heterozygous mutations in the UCP3 gene (V56M, A111V, V192I, and Q252X) in children was associated with dyslipidemia and lower insulin sensitivity [42]. Nevertheless, some limitations exist in our study that are to be addressed in the future. These are:

(i) Mice habitual activity levels have not been evaluated. This is known to influence parameters, such as energy efficiency, body weight gain, and lipid accumulation. Thus, at the moment, it is unclear whether mouse activity levels were altered between gene and or diets, or whether activity levels contributed to study outcomes;
(ii) Whole animal metabolic rate, respiratory quotient, mitochondrial functionality, and WAT lipolysis have only been evaluated in mice that were fed a standard diet. Thus, the estimation of the impact of high fat diet feeding on the above parameters would allow for better clarification of how they are influenced by different diet regimen and the eventual existence of an interaction diet-genotype;
(iii) The evaluation of lipid serum levels, not detected in the present paper, would have provided additional knowledge regarding the role played by UCP3 in influencing lipid metabolism.

## 5. Conclusions

By carrying out our studies under conditions where thermal stress was eliminated, we have highlighted the protective role of UCP3 against ectopic lipid accumulation in lean tissues. Of relevance, by varying the energy and fat intake through the administration of HFD, the role that is played by UCP3 in contrasting HFD-induced fat accumulation clearly emerged. In addition, results that were obtained from studies performed in animals housed at thermoneutrality could be better translated to humans, which choose to live in a thermoneutral environment.

## Figures and Tables

**Table 1:** Body weight, body composition and energy content of each gram of animal weight detected in wild type (WT) and knockout mice (KO) mice at weaning (representing the beginning of the dietary treatment, i.e., time 0 of the experimental procedure).

	WT Time 0	KO Time 0
Body weight (g)	13.6± 0.9	11.5 ± 0.8
Body composition Water (%)	65 ± 1.7	67 ± 1.5
Lipids (%)	13.8 ± 0.3	12.7 ± 0.9
Proteins (%)	16.5 ± 2.4	12.8 ± 2.6
Energy content/g of animal	8.9 ± 0.5	7.64 ± 0.5

Values represent mean ± SE of 5 different animals.

**Table 2:** Body weight, body weight gain, food intake, body rough energy efficiency and visceral WAT weight detected in WT and KO mice, housed at thermoneutrality, and fed either a standard low fat diet (STD) or a high fat diet (HFD) for 80 days, since weaning.

	WT-WT	KO-STD	WT-HFD	KO-HFD
Initial weight (g)	11.88 ± 1.1 ^a^	10.3 ± 1.01 ^a^	14.50 ± 1.2 ^a^	11.14 ± 1.2 ^a^
Final weight (g)	32.4 ± 1.1 ^a^	29.3 ± 1.1 ^b^	38.44 ± 0.6 ^c^	38.98 ± 1.2 ^c^
Body weight gain (g)	19.0 ± 1.4 ^a^	19.0 ± 1.1 ^a^	23.94 ± 1.0 ^b^	27.79 ± 0.94 ^b^
Food intake (kJ)	3294 ± 85 ^a^	3264 ± 107 ^a^	4107 ± 72 ^b^	3938 ± 96 ^b^
Body rough energy efficiency [Body weight gain (g)/food intake (MJ)]	6.33 ± 0.35 ^a,b^	5.86 ± 0.24 ^a^	5.85 ± 0.29 ^a^	7.08 ± 0.29 ^b^
Visceral WAT (g)	2.01 ± 0.14 ^a^	1.36 ± 0.08 ^b^	3.67 ± 0.16 ^c^	3.40 ± 0.24 ^c^
WAT weight/body weight * 100	6.09 ± 0.28 ^a^	4.62 ± 0.17 ^b^	9.51 ± 0.31 ^c^	9.09 ± 0.67 ^c^

Values represent mean ± SE of 7–8 different animals. Differences were evaluated for statistical significance by two-way ANOVA followed by a Tukey’s post hoc multiple comparison test. Different letters indicate that differences between mean values are statistically significant, with *p* < 0.05. Two-ways ANOVA test results: Body weight gain: diet effect *p* < 0.0001, genotype effect ns interaction ns; body rough energy efficiency: diet effect ns, genotype effect ns. interaction *p* < 0.0089; Visceral WAT: diet effect *p* < 0.0001, genotype effect *p* = 0.0094, interaction ns; WAT weight/body weight * 100: diet effect *p* < 0.0001, genotype effect *p* = 0.0223, interaction ns.
